# Gastric Intestinal Affections

**Published:** 1894-03

**Authors:** 


### Gastric Intestinal Affections.

Prof. Germain See (*La France Medicale*, No. 52, 1893) claims that a third of those patients who are thought to be dyspeptics from a stomachic affection are really suffering from an intestinal disease. This is especially true of women, and careful examination reveals no sign of any dilatation, for which they have been treated in vain for years, while the chemical tests are negative. These dyspeptics are not suffering from a gastric disease, for their whole trouble lies in the intestine. The large intestine is the seat of the disease, which deserves the name of muco-membranous enteritis. The small intestine, on the contrary, is in the same condition as the stomach. This muco-membranous enteritis is characterized by disturbances in the function of the colon, gaseous fermentation, dilatation of this viscus, but nothing reveals it with certainty except the passage of mucous, glairy, ribbon-shaped or cylindrical masses with the feces, often unperceived. These mucous products are also accompanied by the hardened residue of undigested food. Treatment consists in evacuating by mechanical means, as olive oil, senna, but not by purgation; calming the pains by the bromides of calcium, strontium or cannabis indica, not by opium. Limit the fermentation and the formation of gas and putrefaction by the phosphate, the salicylate or the biborate of soda in combination, not by benzonaphthol. The diet is that of a normal person, it being only restricted by the presence of habitual constipation or incidental diarrhœa. In general, hearty meat foods—ham, pork, game, calf, boiled eggs—are easily digested, while milk is difficult to tolerate. Potatoes, either mashed or boiled, as well as rice, are well borne, while fruits are of no advantage. Water and tea may be allowed, but no other beverages. Gaseous waters are not permissible on account of the presence of carbonic acid. Alcohol being absorbed in large quantities by the stomach, it is best left alone except temporarily, when digestion is bad. Then a hot sling may be drunken, but red or white wines are not permissible.

*The Cincinnati Lancet Clinic.*

